# Machine learning for a combined electroencephalographic anesthesia index to detect awareness under anesthesia

**DOI:** 10.1371/journal.pone.0238249

**Published:** 2020-08-26

**Authors:** Moritz Tacke, Eberhard F. Kochs, Marianne Mueller, Stefan Kramer, Denis Jordan, Gerhard Schneider

**Affiliations:** 1 Department of Anesthesiology, Klinikum rechts der Isar, Technische Universität München, Munich, Germany; 2 Department of Pediatric Neurology, Munich University Children’s Hospital, Ludwig-Maximilans-Universität München, Munich, Germany; 3 Institute for Computer Science, Technische Universität München, Munich, Germany; 4 Department of Information Systems, Institute for Computer Science, Johannes Gutenberg-Universität Mainz, Mainz, Germany; 5 University of Applied Sciences and Arts Northwestern Switzerland, Muttenz, Switzerland; University of Bern, University Hospital Bern, SWITZERLAND

## Abstract

Spontaneous electroencephalogram (EEG) and auditory evoked potentials (AEP) have been suggested to monitor the level of consciousness during anesthesia. As both signals reflect different neuronal pathways, a combination of parameters from both signals may provide broader information about the brain status during anesthesia. Appropriate parameter selection and combination to a single index is crucial to take advantage of this potential. The field of machine learning offers algorithms for both parameter selection and combination. In this study, several established machine learning approaches including a method for the selection of suitable signal parameters and classification algorithms are applied to construct an index which predicts responsiveness in anesthetized patients. The present analysis considers several classification algorithms, among those support vector machines, artificial neural networks and Bayesian learning algorithms. On the basis of data from the transition between consciousness and unconsciousness, a combination of EEG and AEP signal parameters developed with automated methods provides a maximum prediction probability of 0.935, which is higher than 0.916 (for EEG parameters) and 0.880 (for AEP parameters) using a cross-validation approach. This suggests that machine learning techniques can successfully be applied to develop an improved combined EEG and AEP parameter to separate consciousness from unconsciousness.

## Introduction

Among the criteria of general anesthesia are absence of consciousness and recall. So far, standard monitoring of anesthesia is based on drug concentrations and unspecific effects, e.g. heart rate. It has been suggested that monitoring should evaluate the target organ of anesthesia, the brain. The spontaneous electroencephalogram (EEG) and auditory evoked potentials (AEP) have been used to monitor anesthesia [1–14]. As real-time interpretation of the “raw” EEG is difficult, quantitative analyses to derive numerical values (signal parameters) are required to establish the EEG or AEP as an online monitor. Such monitors have entered clinical practice, and advantages compared to routine care has been shown [15, 16]. There are hints that some of the commercially available methods might be of limited use in the presence of neuromuscular blocking agents [17]. There is therefore need for improved monitoring methods. A combination of several signal parameters that reflect different aspects of brain dynamics to a single indicator could be expedient, because each parameter may contribute additional information associated with the anesthetic drug effect.

Several single parameters, and modifications thereof, have been described. With many parameters at hand, the vast number of possible combinations prohibits evaluations of all different subsets. Furthermore, it is not obvious how to combine parameters in a reasonable way. Some ways to do so are the use of logistic regression [12] or fuzzy inference [5, 6]. The computer science field of Machine Learning offers methods to cope with high-dimensional, noisy data for classification and regression [18–21]. Such methods are based on various approaches, ranging from statistically motivated methods to algorithms mimicking biological systems. This seems to be promising to integrate and evaluate EEG and AEP parameters in monitoring anesthesia.

This investigation evaluates machine-learning-derived composite indicators. Signal analysis is based on data immediately before and after loss and return of consciousness from a EEG/AEP database recorded during a patient study [12]. The hypothesis to be evaluated is that machine learning approaches are able to combine different EEG parameters in a sophisticated way that improves the performance when trying to separate consciousness from unconsciousness. The study could show that this approach is feasible. Conclusive tendencies in the performance measurements show that the method presented here is promising.

## Materials and methods

### Protocol design and data collection

The data used were collected during a randomized, controlled clinical study in 40 adult patients (ClinicalTrials.gov identifier NCT01720615) [12]. The trial had been approved by the ethics committee of the Technische Universität München, Faculty of Medicine, Munich, Germany (Protocol No. 461/01, Chair: Prof. Dr. A. Schömig) on February 14th, 2001. All patients gave written informed consent, none suffered from medical conditions which would prohibit their participation, including contraindications to the used drugs, a history of psychiatric or neurological disease, the use or abuse of drugs affecting the central nervous system, pregnancy, or indication for rapid sequence induction.

All patients were scheduled for elective operations with an American Society of Anesthesiologists physical status of I or II. Consciousness and unconsciousness were defined by the ability of the patient to squeeze the hand of the investigator upon request. These requests were iterated every 30 seconds. The anaesthesia was initiated by an infusion of remifentanil (0.2 μg / kg ⋅ min) followed by either sevoflurane or propofol (0.7 mg/kg, followed by 20 mg every 30 s) until the first loss of consciousness (labeled “LOC1”) occurred, i.e. the patient stopped squeezing the hand of the investigator. Then, the depth of anaesthesia was increased, the forearm of the patient was disconnected from the circulation using a tourniquet, succinylcholine (1.0 mg/kg) was applied, and the trachea was intubated. Afterwards, the administration of propofol or sevoflurane was stopped until the patient started again to follow the requests to press the hand, indicating the first return of consciousness (ROC1). Now, sevoflurane inhalation (5 vol%) or propofol bolus injections (20 mg every 20 s) were restarted. After the second loss of consciousness (LOC2), the tourniquet was removed, anaesthesia was deepened following clinical practice, and surgery was performed. At the end of surgery, patients were again requested to press the investigator’s hand, the first successful request was labeled as second return of consciousness (ROC2). A detailed description of the study protocol is given in the original publication [12].

The study period was from induction (including the clinical event loss of consciousness 1, LOC1) of anesthesia to emergence (return of consciousness 2, ROC2) and included a period of responsiveness after intubation under neuromuscular blockade (ROC1 and LOC2). After the operation no patients remembered this period. EEG and AEP data were recorded using a specialized device [5, 22]. The setup of the EEG electrodes and the configuration of the AEP device have been described previously [12]. Four electrodes were used, placed in the left temporal region (AT1), on the right mastoid (M2), in the central frontal (Fpz, as reference electrode) and in the left frontal area (F7, used as ground). The channel for the EEG parameters was AT1-Fpz while the AEP source data was recorded on M2-Fpz. Recording took place at a sampling rate of 1 kHz using band-pass filter between 0.5 and 400 Hz.

### EEG and AEP parameter derivation

EEG and AEP parameters were used as input for the machine learning algorithms presented below. EEG parameters based on the spectrum of the signal (Weighted Spectral Median Frequency (WSMF) [8], a quotient of WSMF (qWSMF) [6], Spectral Entropy (Sen) [4, 10], Hurst Exponent (HEx) [23] were considered, as well as nonlinear estimations: Approximate Entropy (ApEn) [1], Lempel-Ziv Complexity (LZc) [24] and Permutation entropy (PeEn) [25]. Different AEP parameters were calculated based on the mid latency components of AEPs, where wavelet analysis was used allowing time-frequency resolution of the specific AEP waveform [12, 26]: Wavelet coefficients, amplitudes and latencies of wavelet coefficients, signal energies based on wavelet coefficients, maximum amplitude of retransformed AEPs and variance of the second derivative of wavelet coefficients. Values of 23 EEG and 80 AEP parameters were calculated for every data segment. The duration of the data segments used for the calculation of the EEG parameters was uniformly 10 s; for the AEP parameters, the segments were of variable length, for details please refer to the original publication [12].

### Data analysis

**Fig 1:**
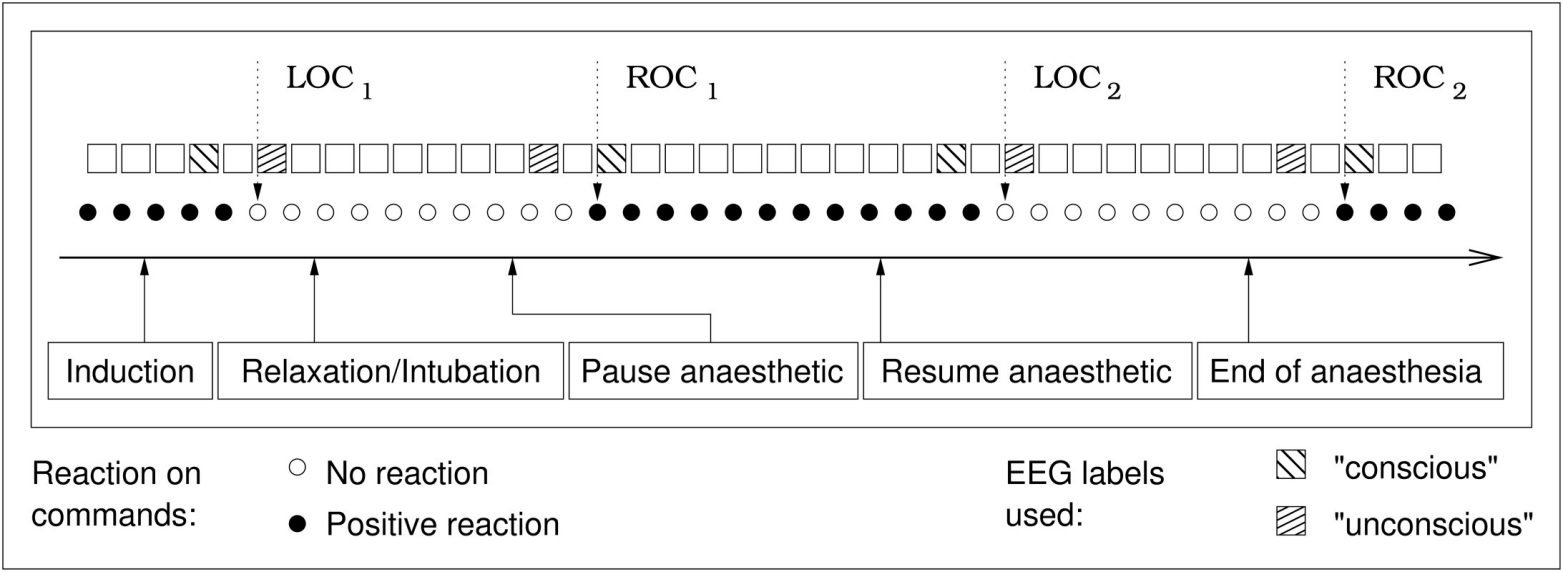
Schematic overview of the study protocol. LOC: Loss of consciousness; ROC: Return of consciousness. Time frame duration: 10s for EEG parameters, variable for AEP parameters. Duration between two requests to push the hand: 30s. The actual surgery period is not depicted in this figure, it took place between LOC_2_ and ROC_2_.

EEG signals of the 40 study patients were analyzed at every clinical event LOC1,2 and ROC1,2 (see Fig 1). The data points were selected in a way that the current state of consciousness was not in doubt. For a “loss of consciousness”, the data labeled “conscious” was recorded before the last successful request to squeeze the hand of the investigator. For the “unconscious” data point, the signals that were recorded after the patient failed for the first time to squeeze the hand were used. Data from the gray area between the last successful request to squeeze the hand and the first failure to follow this request were ignored. The same, in the opposite order, holds true for the “return of consciousness” events. Signal analysis is therefore based on EEG signals preceding or following the transitions between consciousness and unconsciousness. This leads to a maximum of 320 data points in total, 8 per patient, four of them labeled “conscious” and four labeled “unconscious”. Data points with and without neuromuscular blockade were used indiscriminately and without regard to the anaesthetic agent used.

There was no manual post-processing of the data. Automatic artifact detection was used to exclude signals of constant amplitude (flat line), values exceeding the measuring range of 250 μV, and rapidly changing signals (amplitude changes exceeding 140 μV/s). For individual parameters the settings of the filters could be modified, e.g. applying a 25 Hz high-pass-filter before AEP averaging.

The assessment of classification results was done using prediction probability (P_K_) [27]. P_K_ is established in anaesthesia to asses the capability of a parameter to distinguish between different anaesthetic levels. A P_K_ value of 1 gives a completely concordant relation between classifier and observed clinical state, 0 implies completely discordant relation and 0.5 means that there is no relation.

In order to evaluate a machine learning algorithm, the algorithm is trained using a “training set” of data and evaluated on a “test set”. To measure the performance in a realistic fashion, data points must not occur both in the training and in the test set [28]. In the present case, this condition has been tightened: To avoid the classification of one data point of a patient using other measurements from the same patient, the data was split patient-wise. For the evaluation of a given classifier, cross-validation with the leave-one-out principle has been used: In every cycle, the training set comprised data from all patients except one, while the measurements from the excluded patient were classified. Repeating this procedure for every patient resulted in a complete set of classifications. Finally, the P_K_ of each patient was derived, and average and standard deviation of the P_K_ values were calculated.

As this study compared the performance of a multitude of classifiers on a large number of different parameter selections, no tests for statistical significance were performed in this part of the study due to the necessary correction for multiple testing and the small size of the data set.

Therefore, for a final evaluation of the used algorithms, the data of 10 patients were held back, leaving a working set of 29 patients. This was done in order to reduce the risks inherent in multiple testing. In the final evaluation, the P_K_ value was calculated over the full test set. In order to estimate confidence intervals for the P_K_, a bootstrap sampling method was employed [29].

### Classification algorithms

In the field of machine learning, a multitude of classification algorithms has been described. It is not obvious which algorithm performs best on a given task. Therefore, several classification algorithms and parameterizations were applied, being support vector machines (with polynomial, linear, sigmoid and radial basis function (RBF) Kernels), a decision tree learning algorithm, a neural network, logistic regression and Bayesian classifiers.

Support vector machines (SVMs) [21] are mapping the training instances to a higher-dimensional vector space using kernel functions. They continue by searching for a hyperplane separating the classes. SVMs can be tuned using several variables, some of them kernel-specific. Describing them would exceed the scope of this publication. For the given task, the C-values used were 0.01, 0.1, 1.0, 10.0 and 100.0 (where for each SVM type only the results using C value with the highest performance were reported), the gamma-value, if applicable, (1/n) with n being the number of input parameters. The degree of polynomial kernels was set to three.

Decision tree learning [20] algorithms calculate decision trees, comparable to the flowcharts found in medical literature. The decision tree learner used in this project was J48, an implementation of the C4.5 algorithm.

Artificial neural networks [18] implement a imitation of the neuronal networks found in the central nervous system. They consist of simple processing elements (or “neurons”) and their connections. The output behavior of these elements is trained according to their inputs. Finally, in a classification task, the input of a neural net is the instance for classification, and the output consists of the class label.

Logistic regression [19] is a kind of regression where the dependent variable is a dichotomy, i.e. there are only two possible values for this variable.

In general, the data used as an input for machine learning algorithms consists of samples from a probabilistic distribution. Bayesian classifiers [28] construct explicit hypotheses about this distribution. They use assumptions about the dependencies between the variables. The “Naive Bayes classifier” is based on the (usually incorrect) assumption that all the input variables are mutually conditionally independent, given the class, therefore reducing the complexity of the problem. “Bayesian Net” classifiers employ a more sophisticated way to model the dependencies between the variables using a graph.

The libSVM 2.84 (https://www.csie.ntu.edu.tw/cjlin/libsvm/) package was used for the SVMs, all the other algorithms were implementations from the weka 3.4.4 (https://www.cs.waikato.ac.nz/ml/weka/) package.

### Feature selection

Generally, the number of features (EEG and AEP parameters) used as input for a learning algorithm is not limited. Here, this offers the option to calculate a maximum number of EEG and AEP parameters and to use all of them. There are reasons to select only a subset of the available features. These include: (I) The calculation and classification of additional data does not come for free. A reduction of the data needed for classification leads to a reduction of the resources needed to collect and classify them. (II) The contamination of the data by inserting features that are irrelevant with respect to the target variable increases the risk that the classification algorithms base their hypotheses on noise within the data that, by chance, perfectly separates the training set data. This “overfitting” of classifiers to the training data leads to reduced performance on real-world data. (III) The selection of some features while disregarding others can be used as a quality criterion for the features and therefore help to direct further research. (IV) The higher the count of features included into an index, the higher the risk of artifacts in one of the features, leading—depending on the method to combine them—to an unusable indicator value.

Therefore a feature selection algorithm [30] was used in this investigation. The algorithm analyzes the data to estimate the value of every parameter and sorts the parameters accordingly. The result of this feature selection process is therefore a list of features ordered by their utility value.

In the present case, the utility function used was the “information gain” [20], basing on information entropy. This is a measure of the purity of a set of samples; a pure sample has entropy of 0, while the maximum entropy is dependent of the number of different elements in a sample. The information gain of a feature is the expected reduction of entropy over the data if it is split using a threshold on this feature.

## Results

The presentation of the results consists of three parts: After the presentation of the data set characteristics, the performance of the different classification algorithms on the selected parameters will be presented. Then, the influence of modifications to the input data is evaluated.

### Data set characteristics

Originally, the EEG of 40 patients with 8 data points each were available, leading to a data set 320 data points. Artifact filtering lead to removal of 41 items such that 279 signals (133 “conscious”, 146 “unconscious”) were used for the analysis. For one patient all data points were removed. Therefore the measurements from 39 patients were used.

### Classifier performance

The feature selection was used to provide the input parameters of the classification algorithms. Every tested classifier was successively applied on the n parameters with the highest utility values, where n ranged from 1 to 20.

Table 1 shows for every classification algorithm the highest results obtained by the leave-one-out evaluation over the reduced data set. The first column shows the name and parameters of the algorithm, the second one the highest average P_K_ value achieved by this algorithm, and the third one the number of parameters that yielded the optimal performance. SVM is the algorithm leading to highest P_K_ over all the other evaluated algorithms. The non-SVM classifier with highest P_K_ is the naive Bayes algorithm using kernel estimations for modeling numeric attributes, listed as “NaiveBayes -K”.

**Table 1:** Highest prediction performances of the different classifiers using plain information-based feature selection, along with the number of parameters used in the best prediction run. For comparison, the performance of the single parameter with the highest PK value, permutation entropy, is given.

Classifier	Performance P_K_ ±*SD*	Number of parameters
SVM, Polynomial, C = 100	0.935±0.11	12
SVM, Sigmoid, C = 10	0.932±0.11	19
SVM, RBF, C = 1	0.932±0.11	19
SVM, Linear, C = 0.1	0.931±0.10	12
NaiveBayes -K	0.922±0.11	10
NaiveBayes	0.919±0.11	5
Logistic Regression	0.912±0.13	12
Multilayer Perceptron	0.894±0.13	2
Bayes Net	0.885±0.12	11
J48	0.802±0.19	13
For comparison: PeEn	0.913±0.114	1

P_K_: prediction probability, SD: standard deviation, SVM: support vector machine, RBF: radial basis function, J48: Name of a decision tree learning algorithm, PeEn: Permutation entropy.

Table 1 also shows that the SVMs perform best using higher number of parameters; the other algorithms reach their performance optima with parameter counts around ten. But, as Table 2 shows, when all the classifiers were applied to ten parameters, the SVMs still show the highest prediction probability.

**Table 2:** Performance of the learning algorithms on data sets with the 10 parameters with the highest information gain. The single parameter with the highest PK value, permutation entropy, is given for comparison.

Classifier	Performance P_K_ ±*SD*
SVM, Polynomial, C = 100	0.928 ± 0.11
SVM, Sigmoid, C = 10	0.926 ± 0.11
SVM, RBF, C = 1	0.925 ± 0.11
NaiveBayes -K	0.922 ± 0.11
SVM, Linear, C = 0.1	0.921 ± 0.11
NaiveBayes	0.907 ± 0.12
Logistic Regresion	0.893 ± 0.13
Multilayer Perceptron	0.864 ± 0.19
Bayes Net	0.852 ± 0.16
J48	0.770 ± 0.19
For comparison: PeEn	0.913 ±0.114

P_K_: prediction probability, SD: standard deviation, SVM: support vector machine, RBF: radial basis function, J48: Name of a decision tree learning algorithm, PeEn: permutation entropy.

**Fig 2:**
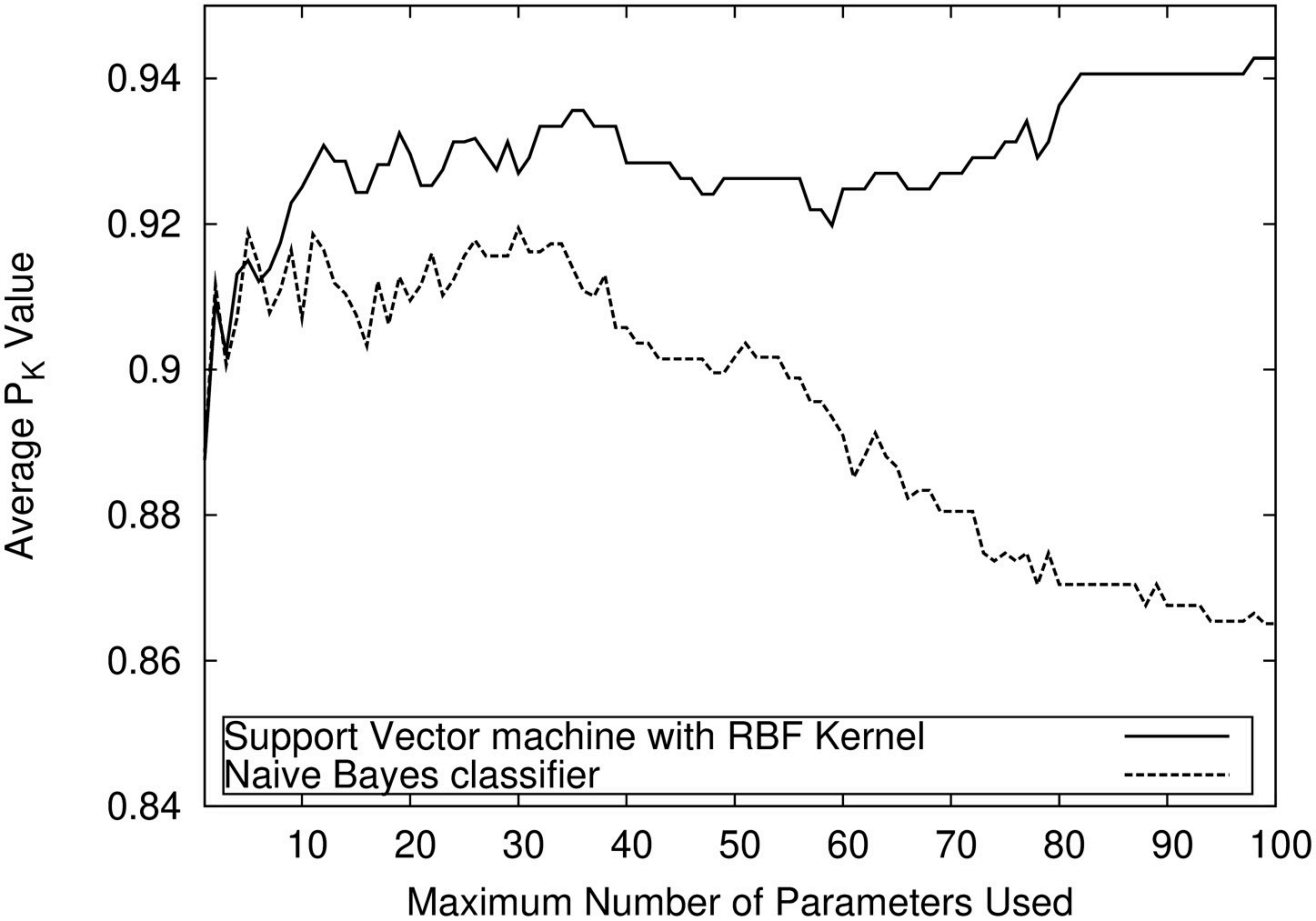
High-dimensionality performance. Performance comparison on large parameter sets between a SVM and a Naive-Bayes Classifier. The SVM is able to tolerate high-dimensional data input, while the performance of the Naive-Bayes-Classifier is decreasing.

To illustrate the performance of the classifiers on large parameter sets, Fig 2 shows the average P_K_ values of an SVM with RBF kernel and of the naive Bayes classifier on parameter sets with 1 up to 100 parameters. The SVM is better over nearly the entire spectrum and its performance increases with the number of parameters, where the naive Bayes classifier worsens.

Finally the performance of a compound indicator based on these results was compared to single parameters. In order to derive statistically valid values, this investigation used the data from the ten held-back patients as a test set. P_K_ values were computed on full-size (i.e. in this case the data from ten patients) data samples. Confidence intervals were calculated using a bootstrap resampling method. The chosen indicator was a SVM with a RBF kernel, using 20 parameters identified by the feature selection on the training set data. The single parameters were the AEP parameter and the two EEG parameters with the highest P_K_ values. Table 3 shows the results of this proceeding. In contrast to the previous evaluations, the compound indicator reaches lower values than one of the single parameters. These results are statistically not significant as demonstrated by the huge overlap of the confidence intervals. An additional, analogous run on a different random patient sample with size ten produced entirely different results. Here, the mean P_K_ values (in the same order as in Table 3) were 0.840, 0.836, 0.784 for the single parameters as well as 0.844 for the compound indicator.

**Table 3:** Performance results of three single parameters as well as one compound indicator, being a SVM with an RBF kernel using 20 parameters. PK analysis was performed using a bootstrap evaluation based on the data from the ten held back patients (test set).

Parameter / Classifier	P_K_ with 95% confidence intervals
WSMF (fhigh = 49 Hz)	0.910 (0.822 to 0.977)
PeEn (fhigh = 49 Hz)	0.935 (0.859 to 0.990)
maximum amplitude of retransformed AEPs of wavelet level D3	0.860 (0.764 to 0.940)
Compound indicator	0.921 (0.764 to 0.940)

fhigh = cutoff frequency of the low-pass filter, P_K_: prediction probability, PeEn: permutation entropy, WSMF: weighted spectral median frequency, AEP: auditory evoked potential.

### Data selection results

**Fig 3:**
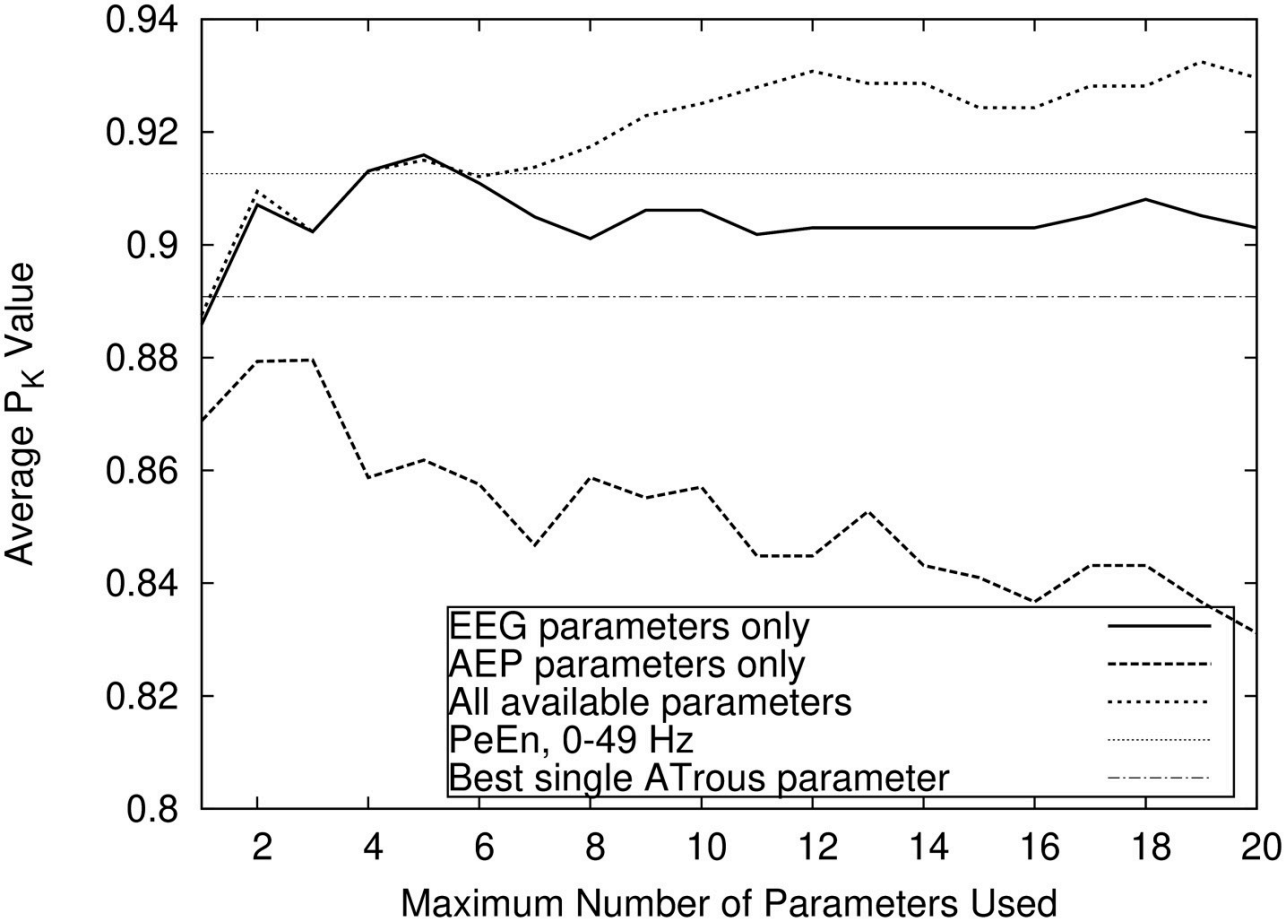
Pure EEG vs pure AEP indicators. Comparison of the prediction performances using the RBF-Kernel SVM reached by allowing only either EEG or AEP parameters, or combinations of both. The single EEG and AEP parameters with the highest P_K_ are shown for comparison.

**Fig 4:**
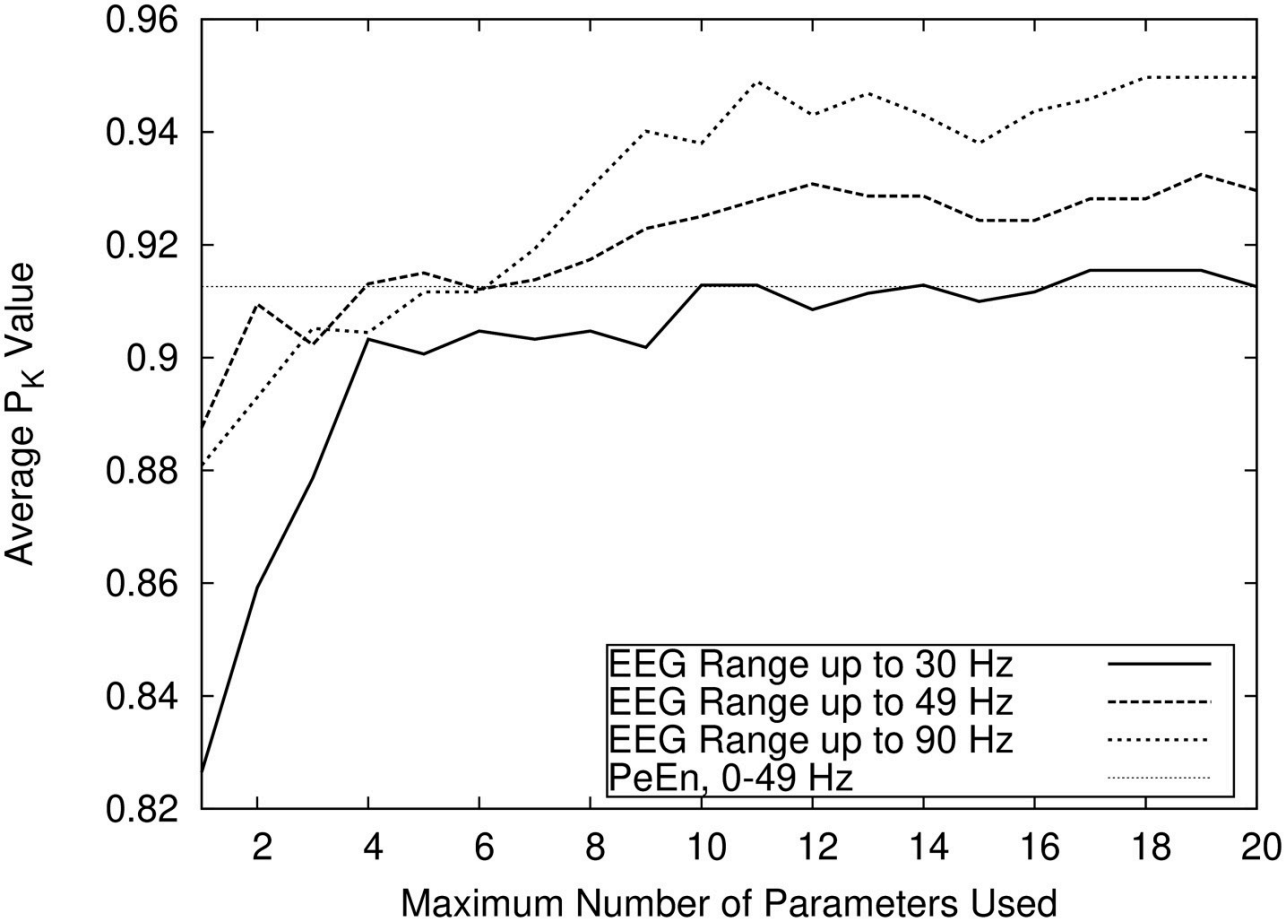
Influence of the EEG low pass filter setting on prediction probability. Comparison of the influence of the EEG low pass filter settings (fhigh) on prediction performance. The figure shows that an increase in the EEG signal range leads to an increase in prediction accuracy. The single EEG parameter PeEn (permutation entropy) is shown for comparison.

As shown in Fig 3, the combination of EEG and AEP parameters outperforms both “pure” parameter sets. The figure additionally shows (as horizontal lines) the performance of two single parameters, in this case the EEG and AEP parameters that, taken alone, yield the highest PK value. The maximum PK values obtained using compound indicators combining only AEP or EEG-Parameters were 0.880 ± 0.14 and 0.916 ± 0.11, respectively. The combination of AEP and EEG parameters yielded a PK of 0.935 ± 0.11. The EEG signal low pass filter settings influence the prediction performance. Fig 4 shows that prediction accuracy increases with the range of the EEG spectrum. The maximum PK value reached using exclusively parameters without the gamma band (i.e. with fhigh = 30 Hz) was 0.915 as opposed to values of 0.935 and 0.950 for fhigh at 49 Hz respectively 90 Hz.

## Discussion

In this investigation, the value of several machine learning classifiers in the interpretation of EEG and AEP data was assessed. Their ability to separate consciousness from unconsciousness during general anesthesia was evaluated. The data was collected before and after, but not during surgery. This might reduce the clinical significance of the results.

A major limitation of the study is the scarcity of the data, which was even increased by the way machine learning analyses must be performed. The common approach in medicine is to analyse all data for a certain end point. The procedures used like e.g. the calculation of a mean are not of specific interest. In machine learning, the data is seen as given, and only the processing is important. The processing step—the “learning”—consumes data. This data must not be used for the final performance measurement. Otherwise, a calculation procedure that simply memorizes the data would have advantages that do not hold in real life. This problem is called “overfitting”. To overcome it, the available data must be split into a training set used for the learning step, and a test set for the evaluation of the so-derived classifier. All attempts to calculate significant differences must be based on the test set. A small test set, as in this case, has the same consequences as a small sample size in a more traditional medical trial.

It was therefore not possible to show any significant advantage of indicators based on several parameters, compared to single-parameter methods. The wide confidence intervals in the final evaluation implied that the test set was too small to derive any statistically significant results. A final evaluation and statistical validation should therefore include additional data.

It can be argued that not only the size of the data set but also the number of the evaluated machine learning algorithms was too small. In fact, there are far more algorithms (e.g. ensemble methods and deep learning approaches) available. The claim of this study was not to identify the universally “best” classifier for the task at hand but rather to demonstrate the feasibility of the approach.

With these limitations in mind, the analyses on the test set itself i.e. excluding the final evaluation on the ten held-back patients) still showed some tendencies that were both consistent and not plausibly linked to overfitting. These tendencies showed an improvement of the classifier’s performance when trained with qualitatively more diverse data. The highest prediction probability was measured using both EEG and AEP parameters. This suggests that both approaches capture independent information regarding the level of consciousness. An extended EEG spectrum with a high cutoff frequency of 90 Hz (compared to a high cutoff frequency of 30 Hz) improved performance. During the test set evaluations, performance was consistently improved relative to the “best” single parameters like permutation entropy; it is noteworthy that these “best” parameters were derived using the present data and, therefore, a bias in favor of these parameters could be suspected.

Furthermore, SVM-based classifiers performed best on all parameter set sizes including very large parameter sets. The highest PK value of a SVM on data sets with at most 20 parameters was 0.935 ± 0.11 compared to 0.922 ± 0.11 as obtained by the best non-SVM algorithm. This statement holds only with respect to the machine learning approaches used in this study. The list is far from being exhaustive, as many other classificators like ensembles or deep-learning approaches were not used. It is noteworthy that the support vector machines were the only algorithms were a limited optimization with respect to the learning settings (by using multiple C values) was applied.

Taken together, the results from this investigation seem to imply that the use of machine learning algorithms with a broad data base can be used to construct an indicator that outperforms every single parameter. Therefore, the implementation of an efficient monitoring device to separate consciousness from unconsciousness during anesthesia based on EEG and AEP parameters which are combined by machine learning classificators seems possible.

## Supporting information

S1 Data (TXT)
